# Design and standard utilization of safety devices for the radiation treatment management of heavy patients

**DOI:** 10.1002/acm2.13616

**Published:** 2022-04-30

**Authors:** David Kanchaveli, Samuel Hellman, Dale Lovelock, Giorgi Archuadze, Cesar Della Biancia

**Affiliations:** ^1^ Department of Medical Physics Memorial Sloan Kettering Cancer Center New York New York USA

**Keywords:** belt, heavy patient, scissor jack, support stand, treatment couch, treatment disruptions, wooden brace

## Abstract

**Purpose:**

Increasing number of heavy cancer patients has created challenges in diagnostic imaging and radiation oncology. Practical weight limits of the equipment can become an obstacle both for imaging and treatment of these patients. Most magnetic resonance imaging and computed tomography (CT) tables’ static load capacities are between 450 and 500 pounds, and linear accelerator tables can support similar weights depending on the type of the table and manufacturer. One recurring issue we encountered was failure of the treatment couch's longitudinal drive belt due to heavy patients’ sudden movement. In several cases, snapping of the longitudinal drive belt occurred when the patient's weight was under 300 lbs (below the rated weight limit). Additionally, we observed vertical deflection of the couch when extended/cantilevered with heavy patients. The purpose of this work was to implement immobilization methods and safety devices for radiation treatment management of heavy patients in order to increase patient/provider safety, prevent treatment couch damage, and reduce treatment disruptions.

**Materials and methods:**

We created three safety devices for treatment management of heavy patients. Wooden brace and Scissor jack were used to lock the couch longitudinal axis (while the couch longitudinal drive was floated) during the setup of a heavy patient and absorb the mechanical impulse applied to the couch longitudinal drive belt. Wooden brace was built in house and positioned in between the wall and treatment couch to lock the longitudinal axis. Commercially available 10 in × 10 in scissor jack lift with adjustable height 3 ½ in – 13 in was modified to increase effectiveness and safety. An additional stand was created with adjustable height and rolling rubber wheels to support the couch when extended/cantilevered with heavy patients.

**Results:**

Using these devices prevented the longitudinal belt from breaking and improved the patient/therapist safety at eight treatment sites within our network. No farther couch belt failures were observed since devices were introduced for clinical use. All three devices can be used and removed without any modifications done to the treatment couch.

## 1 INTRODUCTION

Over the last two decades, obesity rates in the US have increased among adult men and women.^1^ In 2017—2018, the age‐adjusted obesity prevalence among adults in the US was 42.4%.^2^ The radiotherapy treatment of heavy patients can pose risks both to the patient and therapists with the possibility of equipment failure. In particular, the treatment couch's longitudinal drive belt can snap, allowing the couch top with the patient to float unsecured. While placing the patient onto the treatment couch and getting them into the initial setup position, the patient is generally asked to move into the proper position. They may be asked to shift their seated position, which can involve the patient lifting their body off the couch top, moving longitudinally, and then settling into the new position. This movement is not always done smoothly, and the landing may be abrupt. Thus, even if the patient's weight is below the static load limit, the dynamic load caused by the longitudinal shock – or impulse – from the movement may be sufficient to snap the drive belt.

We had several setup events for heavy patients during which the couch longitudinal belt snapped even when the patient's weight was less than 300 lbs, which is well below the rated weight limit of the couch (Table 1). In addition to safety concerns, these failures cause machine downtime of approximately 5 h (replacing the belt and calibrating the couch longitudinal axis). This can lead to a delay in access to care for other patients. Another reason to investigate a more robust solution is that we observed deflection of the couch top when extended/cantilevered near the maximum range. While no mechanical failures were observed, there was concern about the undue stress on the equipment leading to a potential safety hazard as well as concerns about the accuracy of target localization when the patient was positioned on a cantilevered couch. To address this potential hazard and degradation of the accuracy of treatment delivery, we report on our fabrication and implementation of special safety devices to improve patient safety and permit these patients to receive treatment with advanced and accurate localization and delivery techniques.

**TABLE 1:** Varian couch weight limitations^4^

**Couch type**	**Absolute weight limits**
Basic integrated IGRT	500 pounds evenly distributed
Perfect pitch 6DoF	440 pounds evenly distributed
Basic integrated IGRT + kVue	440 pounds evenly distributed
Perfect pitch 6DoF + kVue	330 pounds evenly distributed

## 2 METHODS

### 2.1 Longitudinal bracing

**FIGURE 1:**
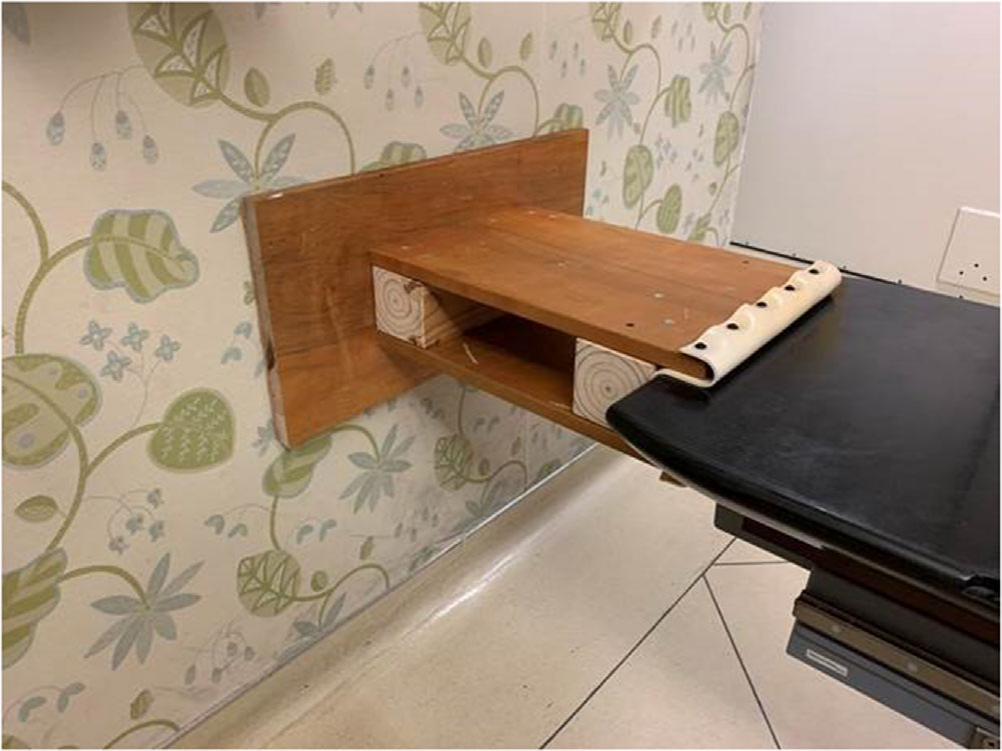
Wooden brace (built by mechanical engineering and instrumentation team Memorial Sloan Kettering Cancer Center (MSKCC) 8/15/2019)

The first solution, which was developed and implemented in August 2019 for an urgent patient treatment situation, involved the use of a padded wooden brace (Figure 1) that was built in‐house. The couch was vertically lowered and then floated longitudinally (the longitudinal drive belt was disengaged). The brace was mounted on the end of the couch and positioned against the nearby wall. The couch was manually pushed against the brace by two therapists positioned at the opposite end of the couch. During patient ingress/egress and during any patient repositioning, the couch was floated longitudinally with two therapists pressing the couch firmly against the brace.

This procedure required four people to get the patient on/off the couch; two therapists to push the couch against the wall, and two to hold the brace against the wall. This initial solution was unique to the treatment room and demonstrates the effort taken to protect the longitudinal drive belt. Unfortunately, this solution did not work in larger treatment rooms where the couch was too distant from the rear wall. This led to the development of a more general solution to improve safety for the patient and the therapist treatment team.

### 2.2 Scissor jack locking device

**FIGURE 2:**
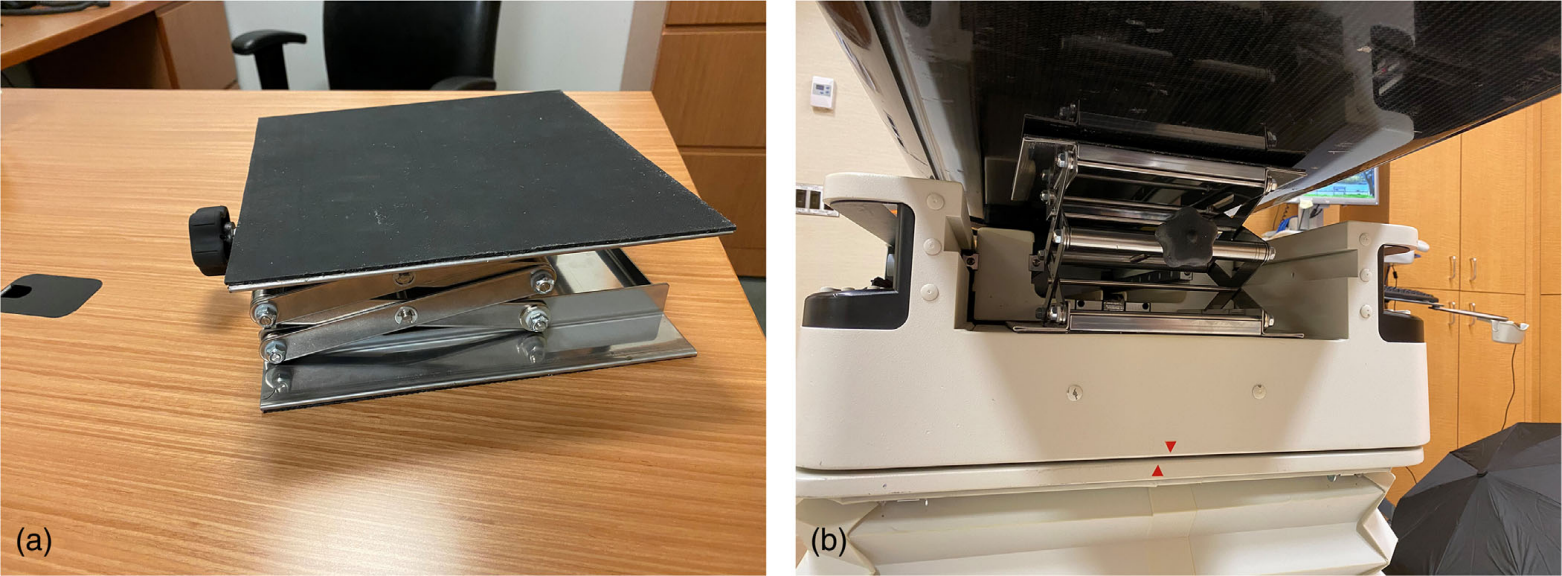
(a) Modified scissor jack. (b) Position of scissor jack underneath the couch top

To develop a robust solution for installation in multiple treatment rooms and centers, a commercially available 10 in × 10 in scissor jack lift^3^ (adjustable height 3 ½ in – 13 in) was modified (Figure 2a) in order to lock the couch longitudinal axis during the patient setup, while the couch longitudinal axis is in floating mode. To increase effectiveness and safety, a 0.125 in thick ethylene propylene diene monomer rubber sheet was affixed to the top and bottom surfaces of the jack, and the height adjustment handle was permanently fixed to the drive screw using a dowel pin. The former modification provides necessary friction to keep the jack in place, and the latter prevents the knob from becoming dislodged or replaced with a larger (higher torque‐generating) knob during use.

The device is placed from the gantry side underneath the couch top (Figure 2b). The scissor jack is then expanded until it is pressing against the underside of the couch top. Once tightened, the jack is lodged firmly between the couch top and frame, which prevents the couch from moving along the longitudinal axis. Because the longitudinal drive belt is disengaged, the impulse generated by sudden movement of a heavy patient is absorbed by the device, and no further bracing or manual handling is necessary.

**FIGURE 3:**
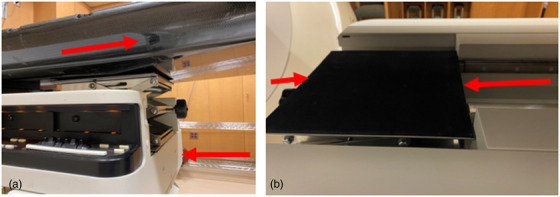
Positioning of scissor jack device

To properly place the device, the longitudinal drive is disengaged, and couch top is moved all the way back. After placing the scissor jack between the couch top and the base frame, as shown in Figure 2a,b, the couch is moved to an appropriate location where the bottom section is flat, and the jack has the largest contact surface (Figure 3a,b).

The scissor jack is expanded by rotating the handle until the couch top is fully engaged. We specifically chose a scissor jack with a maximum lifting capacity of 247 lbs,^3^ which is lower than the specified vertical weight limit (Table 1) of the couch.^4^ We presented the solution to the couch's manufacturer and obtained approval to safely use the device.

### 2.3 Vertical support

**FIGURE 4:**
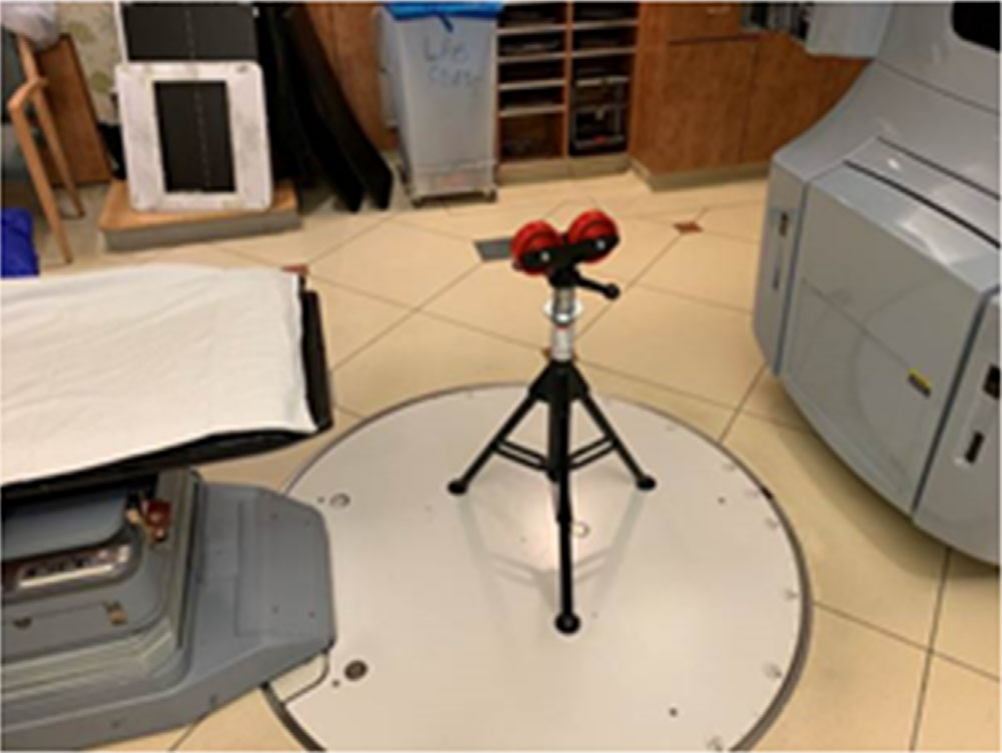
Couch support stand

An additional support stand was built in‐house (Figure 4). This device is an adjustable height stand with rolling rubber wheels to allow the couch top to move while being supported vertically. It is placed under the couch top for cases where the couch needs to be extended significantly beyond the pedestal (for example, pelvis or prostate treatment), and the patient's weight is within 20 pounds of the machine's rated weight limit^4^ (Table 1). The purpose of this device is to add extra vertical support in these cases and prevent deflection and/or possible failure.

## 3 RESULTS AND DISCUSSION

### 3.1 Planning considerations

For patients who were determined at the time of simulation to require the use of the support for treatment, the considerations needed to include consideration of the bore diameter of the computed tomography (CT) Simulator. For example, the outer contour of such patient sometimes had areas (mostly laterally) with missing tissue (after reconstruction of the images). The creation of a reasonable estimate of the patient's body habitus was important when considering eligible beam directions (especially laterally) for treatment. One solution we used to overcome this problem was to estimate the outer contour in the areas of missing tissue and add a structure with hounsfield unit value similar to tissue.

The use of the Couch Support Stand also limited the beam directions (posteriorly) of the treatment fields. Our treatment team found it helpful to do a mock setup using the patient's mold to estimate the isocenter location and verify how far posteriorly the Gantry could safely travel (with generous margins) without hitting the couch and couch support stand. When feasible considering the patient anatomy and the location of the couch support system, we found it preferable dosimetrically to use VMAT, particularly for abdomen and pelvic cases. Treatment planning included a slightly larger margin for the planning target volume due to the lack of volumetric imaging and the residual rotations that were more complicated to fix using the treatment couch positioning.

The methods described above were developed and implemented for 30 linear accelerators at eight treatment sites within our network to support our OneMSK philosophy for patient treatments. When a patient was identified who needed treatment using the couch support stand, the treatment team needed to be aware of the restrictions in the use of posterior gantry angles for both treatment and imaging. Similar limitation has been observed by other authors.^5^ It also eliminated the possibility of cone beam computed tomography imaging due to the lack of gantry clearance. Depending on the site, we advised our therapists to use a kilovoltage radiographic technique far outside of the usual range. For pelvis sites, anterior and lateral kilovoltage peaks were typically 120 and 150, with 120 mAs and 320 mAs, respectively.

The scissor jack or wooden brace devices lock the couch longitudinal axis during the setup of a heavy patient (getting on/off the couch). The device absorbs the mechanical impulse applied to the couch, which prevents the longitudinal belt from breaking and improves the patient/therapist safety. Our experience with the scissor jack device showed that for a perfect pitch couch, the pitch and roll must be zeroed before placing the scissor jack to avoid possible damage to the device. A torque limiter can be added to the height adjustment handle to set the force applied to the couch top. The device was tested in‐house and approved by Varian (manufacturer). It has been in use for the treatment of over 25 heavy patients successfully at MSKCC.

## 4 CONCLUSION

Three safety devices—scissor jack, wooden brace, and support stand—have been tested and successfully used for the treatment of heavy patients in the radiation oncology department. All three devices can be used and removed without any modifications done to the treatment couch. The solutions were presented to the couch manufacturer and approved for clinical use in May 2021. Since the introduction of the above‐mentioned safety devices, patients have been successfully treated without further belt failures.

## AUTHOR CONTRIBUTIONS

*Contributed to the idea, design and implementation of the work, analysis of the results, and the writing of the manuscript*: David Kanchaveli. *Design/idea of the Wooden brace, Scissor Jack and support stand, analysis of the results, and editing/revising of the manuscript*: Samuel Hellman. *Contributed to the imaging consideration part and editing/revising critically for important intellectual content*: Dale Lovelock. *Contributed to implementation of the work, editing/revising of the manuscript*: Giorgi Archuadze. *Contributed to implementation of the work, treatment planning consideration part, and editing/revising of the manuscript*: Cesar Della Biancia.

## CONFLICT OF INTEREST

The authors declare that there is no conflict of interest that could be perceived as prejudicing the impartiality of the research reported.

## Data Availability

Data sharing is not applicable to this article as no new data were created or analyzed in this study.
